# Eight months follow-up of corneal nerves and sensitivity after treatment with cenegermin for neurotrophic keratopathy

**DOI:** 10.1186/s13023-022-02237-5

**Published:** 2022-02-21

**Authors:** Emilio Pedrotti, Erika Bonacci, Chiara Chierego, Alessandra De Gregorio, Tiziano Cozzini, Tommaso Brighenti, Grazia Caldarella, Giovanlorenzo Pastore, Adriano Fasolo, Giorgio Marchini

**Affiliations:** 1 Ophthalmic Unit, Department of Neurosciences, Biomedicine and Movement Sciences, University of Verona, Policlinico G.B. Rossi, P.Le L.A, Scuro 10, 37134 Verona, VR Italy; 2 Ophthalmic Unit, San Bassiano Hospital, Via dei Lotti, 40, 36061 Bassano del Grappa, Italy; 3 The Veneto Eye Bank Foundation, Padiglione G. Rama, Via Paccagnella 11, 30174 Zelarino Venezia, Italy

**Keywords:** Cenegermin, Corneal nerves, Corneal sensitivity, In vivo confocal microscopy, Nerve growth factor, Neurotrophic keratopathy, rhNGF

## Abstract

**Backgroud:**

Cenegermin (Oxervate, Dompè Farmaceutici, Milan, IT), a recombinant human NGF, is a potentially healing new drug for neurotrophic keratopathy (NK), a rare but challenging disease affecting the cornea. To date, studies that evaluate its mid-term effect on corneal nerves and sensitivity are lacking.

**Objective:**

To evaluate the recovery and morphology of subbasal corneal nerves in patients treated with Cenegermin for NK and assess their correlation with corneal sensitivity.

**Methods:**

This prospective, observational case series study was carried out between May 2018 and August 2020 at the Ophthalmic Clinic of the University of Verona. Clinical evaluation, sensitivity, and in vivo confocal microscopy (IVCM) were performed in the central and all four corneal sectors at baseline, the end of therapy (8 weeks), and 2, 4, and 8 months after therapy. Consecutive patients with NK (stage 2–3), treated with Cenegermin (1 drop 6 times daily for 8 weeks), were enrolled. During each visit, Corneal nerve fiber length (CNFL), corneal nerve fiber total branch density (CTBD), corneal nerve fiber fractal dimension (CNFraD) and Cochet-Bonnet esthesiometry (CBE) were measured.

**Results:**

We enrolled 18 patients. Complete NK healing was noted in 14/18(78%) patients after 8 weeks of treatment; then in 14(78%), 15(83%), and 13(72%) patients at 2-, 4-, and 8-months, respectively. Starting at 8 weeks through 4-month follow-up there was progressive improvement in CBE in all corneal sectors (*p* ≤ 0.01), which continued thereafter. There was significant corneal nerve regrowth especially in the peripheral cornea: centripetal progression starting at 8 weeks (CNFL and CNFrad) and significant branching starting at 2 months (CTBD), which continued through to the end of follow up. Sector-coupled IVCM and CBE findings correlated at all evaluations (all r ≥ 0.62 starting at 2 months, with highest values in the peripheral sectors).

**Conclusions:**

After Cenegermin we observed a subbasal corneal nerve regeneration, a recovery of sensitivity and a lasting epithelial healing, suggesting that the effect of its action persists several months after discontinuation in patients with NK.

## Background

Neurotrophic keratopathy (NK) is a rare corneal disorder associated with some extent of trigeminal damage along the path of the fifth cranial nerve, from the brain stem to the corneal nerve endings. Neural damage leads to a reduction or a loss of corneal sensitivity, reduced tear production, and eventually spontaneous epithelial breakdown with impairment of corneal healing [1, 2].

NK has been traditionally regarded as an orphan disease with no effective resolutive treatment. In its early stages, the discontinuation of toxic topical and systemic therapies and the application of preservative-free lubricants are recommended; then bandage contact lens, matrix-regenerating agents and autologous serum may be used. Surgery is reserved for severe cases, in which tarsorrhaphy and amniotic membrane transplantation are generally performed [2]. Since 2009, corneal neurotization has been reported; a surgical re-innervation of the affected cornea by direct re-routing a healthy neighboring sensory nerve (direct re-innervation) or by autologous or allogenic nerve grafting (indirect re-innervation). Despite promising results, these techniques are rather challenging and only few cases have been described to date [3].

A more viable option for patients with NK is represented by the treatment with Cenegermin (Oxervate, Dompè Farmaceutici, Milan, Italy), a recombinant human nerve growth factor (rhNGF) recently approved by the European Medicines Agency for the treatment of recalcitrant moderate-to-severe NK and by the U.S. Food and Drug Administration for all stages of NK. Two registrative trials reported that Cenegermin 20 µg/ml (one drop 6 times daily for 8 weeks) is significantly more effective than placebo to achieve complete corneal healing [4, 5].

These studies did not assess the recovery status of corneal innervation, although an improvement in corneal sensitivity was observed and the need for objective evaluation of corneal nerves recognized [6]. Mastropasqua et al. found a significant increase in subbasal innervation after Cenegermin treatment from baseline to week 4, which remained stable at week 8 [7]. Data on follow up after therapy about corneal healing, sensitivity and nerve regrowth are lacking. We hypothesized that improvement in corneal sensitivity might be associated with the recovery and maintenance of subbasal corneal plexus innervation. Accordingly, the aim of the present study was to document 8-months results, in patients treated with Cenegermin, on the maintenance of a stable corneal epithelium, the subbasal corneal nerve status and its correlation with corneal sensitivity.

## Materials and methods

This prospective, observational case series was carried out at the Ophthalmic Clinic of the University of Verona in compliance with the tenets of the Declaration of Helsinki. Institutional Ethics committee of Verona and Rovigo approval (Protocol No. 73518) and written informed consent from all participants were obtained.

Consecutive patients with NK stage 2 (*moderate*, persistent epithelial defect [PED]) or stage 3 (*severe*, corneal ulcer) based on the Mackie classification [7, 8], were enrolled between May 2018 and August 2020. Patients were unresponsive to classical medical therapy (including preservative free lubricants, therapeutic contact lenses and discontinuation of all preserved drops and medications that can decrease corneal sensitivity) for at least 4 weeks and were treated with Cenegermin 20 µg/ml (Oxervate, Dompè Farmaceutici, Milan, Italy), 1 drop 6 times daily for 8 consecutive weeks. A single-dose preservative free antibiotic (Monofloxofta, BIOOS Italia Srl, Fermo, Italy) was administered qid until complete closure of the epithelial defect, all other ocular therapies that could interfere with the epithelialization and bandage contact lenses were discontinued as indicated in the registrative Phase II study [5]. Exclusion criteria were active infective keratitis, ocular surgery in the previous 6 months, and exposure keratopathy not surgically corrected.

Medical history, clinical findings (slit lamp biomicroscopy, fluorescein staining, and corneal sensitivity) in vivo confocal microscopy (IVCM) data were collected at baseline, at the end of the treatment (8 weeks), and at 2-, 4-, and 8-month follow-up visits. Every evaluation, even if minimally invasive, was avoided during the treatment period to exclude potentially confounding factors, such as a mechanical stress on the immature epithelium intrinsically inducible by the tip of the IVCM. Corneal sensitivity was assessed with a Cochet-Bonnet esthesiometer (CBE) (Western Ophthalmics, Lynnwood, Washington, USA) in the central 4 mm of the cornea and in the four peripheral sectors (inferior, nasal, superior, temporal) of the corneal surface. The nylon filament was applied perpendicularly to the corneal surface and the fiber length was gradually shortened from 60 mm by 5 mm until the patient appreciated corneal touch (60 mm represented normal sensation and 0 represented completely absent sensation). The longest filament length resulting in sensation was verified twice and recorded as the measure of sensitivity. IVCM examination (diode-laser 670 nm, Heidelberg Retinal Tomography III with Rostock Cornea Module, Heidelberg Engineering GmbH, Heidelberg, Germany) was performed to assess the subbasal corneal nerve plexus. CBE and IVCM evaluations were performed by two independent operators to avoid operator bias. A drop of ophthalmic tear gel (Tear Gel carbomer 0.3%, Thea Pharmaceuticals, Clermont-Ferrand, France) was used as coupling medium between the microscope objective lens and the corneal surface, previously anesthetized using a drop of topical lidocaine hydrochloride 40 mg/ml (Alfa Intes Pharmaceuticals, Casoria, Italy). Scans of the central cornea and each of the four corneal sectors were taken using a sequence mode by recording two images per second. To increase repeatability, scans IVCM was performed always by the same operator: scans in the 4 mm central cornea were considered central, while for peripheral sectors scans were taken at the half-way between the center and the limbus. To achieve the best image quality of the nerves, the examiner manually focused the subbasal nerves while scanning, by constantly adjusting the axial depth dial. Only high-quality images that were well-focused with good contrast were selected by an experienced masked operator. Oblique sections were excluded.

The apparent direction of nerve regrowth was recorded by the operator, while five scans from each sector were analyzed with automated ACC Metrics analysis software (Imaging Science and Biomedical Engineering, Version 2, Manchester, United Kingdom) [9–15] to obtain the mean corneal nerve fiber length (CNFL), which is the total length of nerves expressed in mm/mm^2^; the corneal nerve fiber total branch density (CTBD), which is the total number of branch points/mm^2^, and the corneal nerve fiber fractal dimension (CNFraD), a dimensionless parameter that measures corneal nerve structure complexity as a ratio of the change in detail to the change in scale.

### Statistical analysis

Statistical analysis was performed with SPSS (IBM SPSS Statistics for Windows, Version 25.0, IBM Corp, Armonk, New York, USA). The non-normal distribution of all variables was assessed by the Kolmogorov–Smirnov test. Qualitative variables are presented as frequency and percentage. Quantitative variables are presented as median and interquartile range (IQR). Differences in contiguous time intervals of CBE and IVCM values were assessed using the Wilcoxon signed-rank test; a two-tailed *p* value of < 0.05 was considered statistically significant. A bivariate correlation between each IVCM and corresponding CBE result at each time, from the end of the therapy to the 8-month follow-up visit, was assessed with Spearman's rank correlation test. A *p* value of < 0.05 was considered statistically significant (one-tail test).

## Results

The study population was 18 eyes of 18 patients; the median (IQR) age was 61.5 (20.3) years (Table 1).

**Table 1:** Patient demographics and clinical characteristics

Nos.	Sex	Age	Eye	BCDVA (LogMAR)	Systemic disease	Eye disorder/previous ocular surgery	NK stage (Mackie)
1	M	60	OS	0.9	DM2, rheumatoid arthritis	Severe bacterial keratitis followed by PK in 2016, repeated AMTs	2
2	F	79	OS	0.5	SLE	PK for KC in 1976	2
3	M	88	OS	0.6	–	Recurrent HSV keratitis	3
4	F	61	OS	0.8	GVHD	Ocular GVHD, pseudomonas keratitis in 2017, PK, RGTA and AMT for PED in 2017	3
5	M	61	OS	0.4	–	Ocular trauma and PK in 1999, trabeculectomy in 2004	2
6	F	50	OD	0.9	–	Eyelash ablation for trichiasis in 1982, exposure keratopathy surgically corrected in 2002, PK in 2015, AMT in 2016	2
7	F	64	OD	0.8	–	PK in 1988 for KC, exposure keratopathy surgically corrected in 2016	3
8	M	65	OS	0.9	–	Alkali burn in 2017	3
9	F	42	OD	0.6	–	Recurrent HSV keratitis	2
10	F	75	OD	0.8	–	PK in 1991 for KC, repeated PK in 2004, lagophthalmos and exposure keratopathy surgically corrected in 2015	3
11	M	84	OS	0.4	Prostatic hyperplasia, DM2, hyperlipemia	PK in 2018 for total corneal leucoma after Corynebacterium and Staphylococcal keratitis	2
12	M	59	OS	0.5	–	Retinal surgery for RD with 360° endolaser retinopexy in 2018	2
13	F	88	OS	0.4	DM2, hypertension, hyperlipemia	Acoustic nerve surgery with trigeminal damage in 2010	2
14	M	62	OD	0.7	Hypertension	Alkali burn in 2015	2
15	F	55	OD	0.4	–	Retinal surgery for RD with 360° endolaser retinopexy in 2017	2
16	F	77	OD	0.5	–	Two PK performed elsewhere, PK for corneal leukoma and edema in 2018, glaucoma treated with topical therapy	2
17	F	39	OS	0.6	DM2	Retinal surgery with PRP in 2018	2
18	F	88	OS	0.6	–	DSAEK in 2016 for Fuchs keratopathy, recurrent HZV keratitis	2

AMT, amniotic membrane transplantation; BCDVA, best corrected distance visual acuity; DM2, diabetes mellitus type 2; DSAEK, Descemet stripping automated endothelial keratoplasty; F, female; GVHD, graft versus host disease; HSV, herpes simplex virus; KC, keratoconus; M, male; OD, right eye; OS, left eye; PED, persistent epithelial defect; PK, penetrating keratoplasty; PRP, panretinal photocoagulation; RD, retinal detachment; RGTA, matrix regenerating agent; SLE, systemic lupus erythematosus

Complete corneal healing was observed in 14 (78%) patients at 8 weeks (end of treatment), then in 14 (78%), 15 (83%), and 13 (72%) patients at the 2-, 4-, and 8-month follow-up after the end of treatment, respectively. In 3 patients (nos. 10, 13, 14), the treatment led only to a reduction in the epithelial defect but failed to completely resolve it. In these cases, after the treatment, weekly reviews and protection with bandage contact lenses, preservative-free topical lubricants and antibiotics were given, throughout the study period. Relapse was noted in two patients at the last follow-up: in one (no. 3) due to a trigeminal ganglion ablation for post herpetic neuralgia at 7 months after the end of therapy, and in the other (no 4) due to worsening of ocular graft versus host disease (GVHD).

**Fig. 1:**
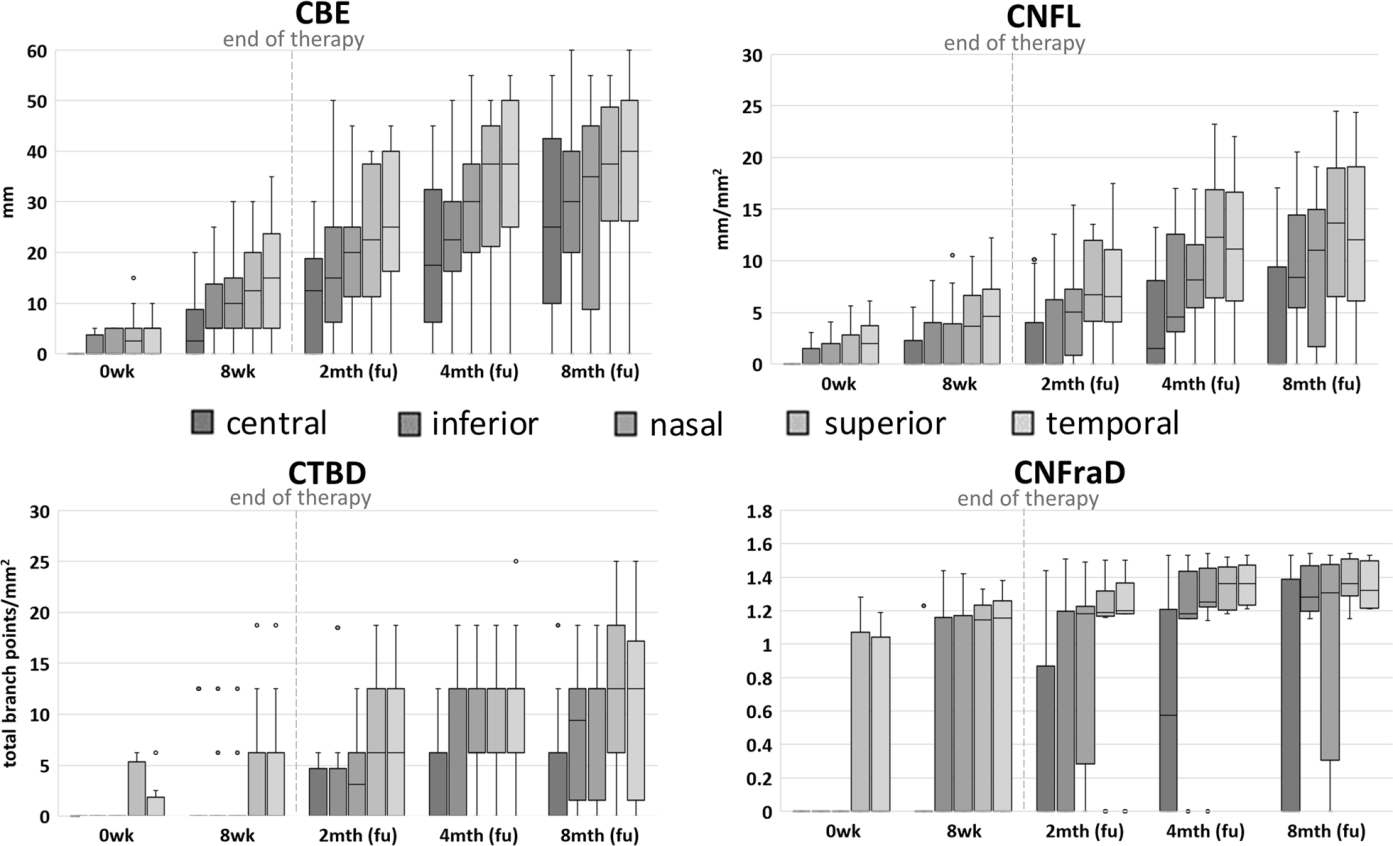
Box and whiskers plots of median CBE, CNFL, CTBD, and CNFraD for each corneal sector at baseline, at the end of therapy, and at each follow-up. There was an overall increasing trend in CBE and IVCM parameters. Boxes represent interquartile range, dots are the outliers, the line in the box is the median value and whiskers represent maximum and minimum values. For *p* values see Table 2

Table 2 and Fig. 1 present the median (IQR) for CBE, CNFL, CTBD, and CNFraD listed by follow-up time and ordered by corneal sector.

**Table 2:** Results of Cochet-Bonnet esthesiometry and in vivo confocal microscopy (IVCM) parameters

	0 wk	8 wk	P (0–8 wk)	2 mth (fu)	p (8 wk–2 mth)	4 mth (fu)	p (2–4 mth)	8 mth (fu)	p (4–8 mth)
	Median (IQR)	Median (IQR)		Median (IQR)		Median (IQR)		Median (IQR)	
CBE									
C	0 (0)	2.5 (8.75)	0.01	12.5 (18.75)	< 0.01	17.5 (26.25)	< 0.01	25 (32.5)	0.05
I	0 (3,75)	5 (8.75)	< 0.01	15 (18.75)	< 0.01	22.5 (13.75)	< 0.01	30 (20)	0.08
N	0 (5)	10 (10)	< 0.01	20 (13.75)	< 0.01	30 (17.5)	< 0.01	35 (36.25)	0.07
S	2.5 (5)	12.5 (15)	< 0.01	22.5 (26.25)	< 0.01	37.5 (23.75)	< 0.01	37.5 (22.5)	0.07
T	5(5)	15 (18.75)	< 0.01	25 (23.75)	< 0.01	37.5 (25)	< 0.01	40 (23.75)	0.15
CNFL									
C	0 (0)	0 (2.30)	0.04	0 (4.03)	0.04	1.51 (8.1)	0.01	0 (9.42)	0.52
I	0 (1.48)	0 (4.02)	0.02	0 (6.26)	0.02	4.53 (9.46)	< 0.01	8.37 (8.96)	< 0.01
N	0 (1.99)	0 (3.89)	0.02	5.06 (6.41)	< 0.01	8.13 (6.11)	< 0.01	11.01 (13.25)	0.11
S	0 (2.82)	3.66 (6.63)	< 0.01	6.74 (7.8)	< 0.01	12.28 (10.46)	< 0.01	13.64 (12.45)	0.06
T	1.99 (3.74)	4.63 (7.26)	0.01	6.55 (7.03)	< 0.01	11.16 (10.51)	< 0.01	12.06 (13)	0.08
CTBD									
C	0 (0)	0 (0)	0.32	0 (4.69)	0.13	0 (6.25)	0.05	0 (6.25)	0.78
I	0 (0)	0 (0)	0.06	0 (4.69)	0.06	0 (12.5)	0.04	9.38 (10.94)	0.01
N	0 (0)	0 (0)	0.06	3.13 (6.25)	0.01	6.25 (6.25)	0.01	12.5 (10.94)	0.32
S	0 (5.31)	0 (6.25)	0.05	6.25 (12.5)	0.02	12.5 (6.25)	< 0.01	12.5 (12.5)	0.26
T	0 (1.88)	0 (6.25)	0.01	6.25 (12.5)	0.02	12.5 (6.25)	< 0.01	12.5 (15.63)	0.67
CNFraD									
C	0 (0)	0 (0)	0.32	0 (0.87)	0.08	0.58 (1.21)	0.01	0 (1.39)	0.64
I	0 (0)	0 (1.16)	0.02	0 (1.2)	0.03	1.18 (0.28)	< 0.01	1.28 (0.27)	0.01
N	0 (0)	0 (1.17)	0.02	1.18 (0.94)	< 0.01	1.25 (0.24)	< 0.01	1.31 (1.17)	0.22
S	0 (1.07)	1.15 (1.23)	< 0.01	1.19 (0.15)	< 0.01	1.36 (0.26)	< 0.01	1.36 (0.22)	0.06
T	0 (1.04)	1.16 (1.26)	0.01	1.2 (0.19)	< 0.01	1.36 (0.24)	< 0.01	1.32 (0.28)	0.27

There was an overall increasing trend for all values and a statistically significant difference between contiguous time intervals (p). Even when the median values were 0, an increase in IQR denoted an increase in the parameters’ values in some patients. 0 wk denotes 0 week (baseline, start of treatment); 8 wk 8 weeks (end of treatment); 2 mth (fu) 2-month follow-up; 4 mth (fu) 4-month follow-up; 8 mth (fu) 8-month follow-up

C, central; CBE, Cochet-Bonnet esthesiometry; CNFL, corneal nerve fiber length; CNFraD, corneal nerve fiber fractal

At baseline, corneal sensitivity was absent in the central corneal sector (the lesion site), while a low sensitivity was present in one or more sectors in 11 patients. CBE showed a significant increase in all corneal sectors at 8 weeks, 2 months and 4 months follow-up (*p* ≤ 0.01) and it improved further at the 8-months follow-up, except in the superior sector, where the median value of CBE was stable. In vivo confocal microscopy (IVCM) revealed centripetal progression of subbasal corneal nerves regrowth, especially in the peripheral cornea. Quantitative analysis of the subbasal nerves images with ACC Metrics showed a significant increase in CNFL in all corneal sectors between each contiguous time interval until the 4-month follow-up (*p* < 0.05). At 8-month follow-up, the CNFL progressively increased in all corneal sectors, except in the central one in which decreased due to the two relapsed patients.

Progressive nerve regrowth was accompanied by a gradual increase in branching over time. A significant increase in CTBD was observed in the superior (*p* = 0.05) and temporal (*p* = 0.01) sectors from 8 weeks, in the nasal sector (*p* = 0.01) from the 2-month follow-up and in the inferior one (*p* = 0.04) from the 4th month. Although no significant branching was detectable in the central corneal sector until the last follow-up, the growing trend of the IQR indicated initial branching also in the central cornea in some patients.

**Fig. 2:**
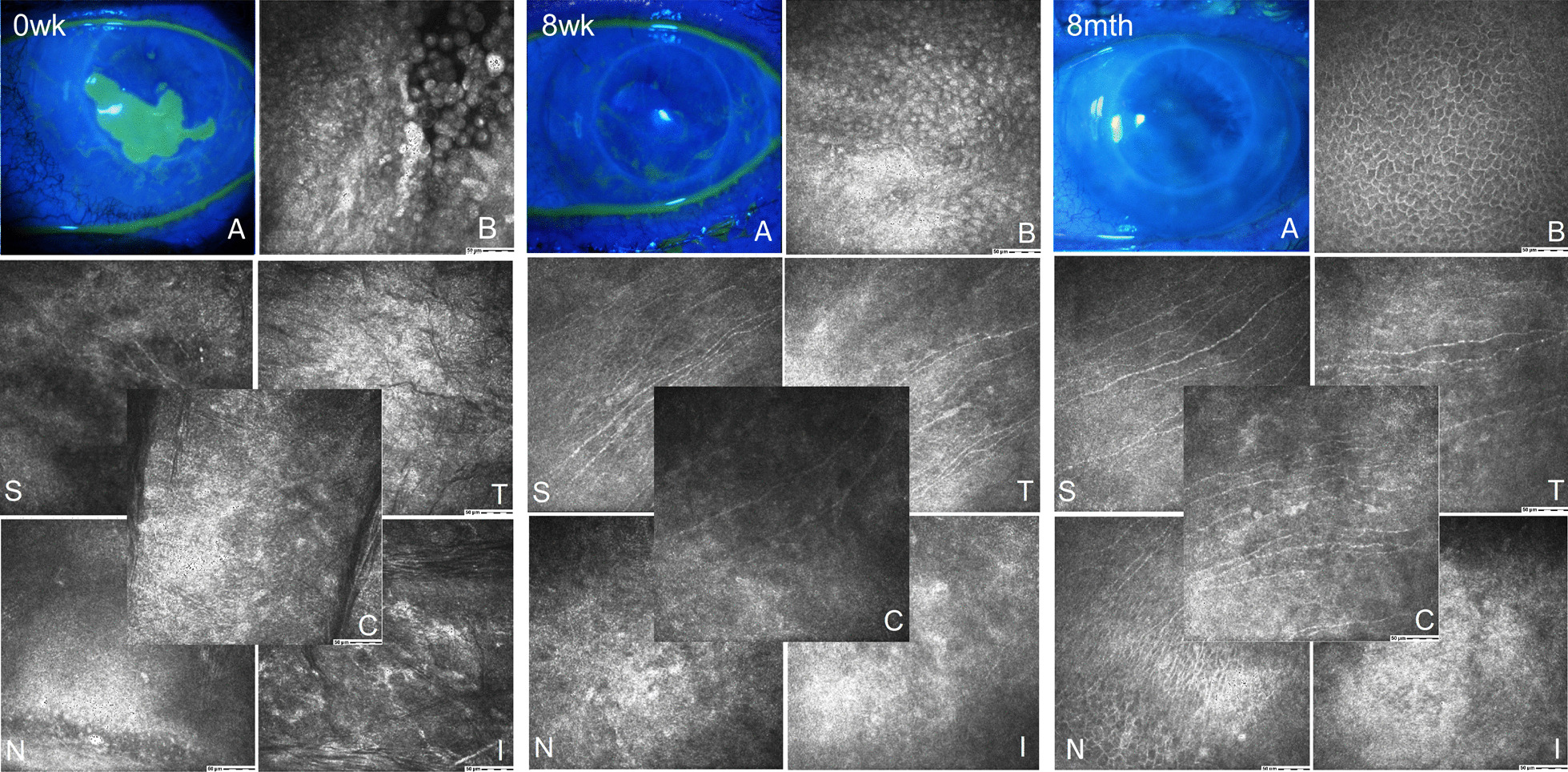
Representative images of fluorescein staining of the corneal surface and IVCM images showing the central and peripheral cornea sectors in a successful case (patient no. 2). 0wk, baseline: corneal ulcer highlighted by positive fluorescein staining (A); presence of loose exfoliating epithelial cells at the ulcer margins (B); rare subbasal nerves visible only in the superior sector (S). 8wk, 8 weeks, end of therapy: complete closure of the ulcer and negative fluorescein staining (A), with immature epithelial cells that fill the bed of the previous ulcer (B); Initial subbasal nerve regrowth from the periphery to the center in the superior (S) and the temporal (T) sector, with rare tiny nerves visible also in the central sector (C). 8mth, 8-month follow-up: absence of fluorescein staining (A); closure of the ulcer is maintained, with presence of mature clear epithelium (B); subbasal corneal nerve plexus present in all but the inferior sectors (T, S, C, N). A, fluorescein staining in cobalt blue light; B, epithelium at the site of the ulcer; C, central sector; I, inferior sector; N, nasal sector; S, superior sector, T, temporal sector

Consistent with the other IVCM parameters, CNFraD significantly increased in all peripheral sectors from the 8 weeks to the 4-month visit (*p* < 0.05), while a significant increase was recorded in the central sector at 4-month follow-up (*p* = 0.01). At the 8-month follow-up there was a further increase in the nasal (*p* = 0.22) and the inferior (*p* = 0.01) sectors, whereas a small reduction was found for the median temporal and central values (*p* = 0.27 and *p* = 0.64, respectively). The latter reduction could be influenced by the two relapsed patients, however the ceiling effect observed in the parameters at the 8 month follow-up could also be related to a longer time elapsed from the cessation of therapy. Figure 2 shows representative images of a successful case at baseline, 8 weeks, and the 8-month follow-up visit.

The Spearman test confirmed a strong correlation between IVCM- and CBE-parameters, reaching statistically significance for all sectors at 8 weeks visit and at every follow-up visit (Table 3). The correlation was not assessable at baseline in some sectors, where all IVCM parameters or all CBE measurements were zero.

**Table 3:** Spearman correlation between ACC metrics parameters and Cochet-Bonnet esthesiometry

	CNFL/CBE					CTBD/CBE					CNFraD/CBE				
	C	I	N	S	T	C	I	N	S	T	C	I	N	S	T
0wk	–	0,61*	0.16	0.49^τ^	0.82*	–	–	–	0.49^τ^	0.59*	–	–	–	0.72*	0.62*
8wk	0.76*	0.82*	0.59*	0.75*	0.87*	0.43^τ^	0.67*	0.57*	0.76*	0.87*	0.43τ	0.82*	0.65*	0.77*	0.86*
2mth (fu)	0.62*	0.75*	0.68*	0.87*	0.87*	0.62*	0.64*	0.67*	0.80*	0.89*	0.62*	0.76*	0.75*	0.76*	0.77*
4mth (fu)	0.75*	0.69*	0.73*	0.88*	0.79*	0.73*	0.72*	0.89*	0.89*	0.71*	0.77*	0.67*	0.79*	0.77*	0.63*
8mth (fu)	0.79*	0.92*	0.87*	0.88*	0.68*	0.79*	0.89*	0.78*	0.82*	0.63*	0.79*	0.94*	0.83*	0.90*	0.71*

A strong correlation between sensitivity and IVCM parameters was found for all sectors from the 2-month follow up onward

C, central sector; CBE, Cochet-Bonnet esthesiometry; CNFL, corneal nerve fiber length; CNFraD, corneal nerve fiber fractal dimension; CTBD, corneal nerve fiber total branch density; I, inferior sector; N, nasal sector; S, superior sector; T, temporal sector; 0 wk, baseline (start of treatment); 8 wk, 8 weeks (end of treatment); 2 mth (fu), 2-month follow-up; 4 mth (fu), 4-month follow-up; 8 mth (fu), 8-month follow-up

(^τ^) value with statistical significance at threshold 0.05 (one-tailed)

(*) value with statistical significance at threshold 0.01 (one-tailed)

## Discussion

This study evaluated recovery, morphology, and maintenance of corneal nerves in patients treated with Cenegermin for NK, and their correlation with corneal sensitivity. After the 8 weeks of treatment and during the follow-up evaluations up to 8 months after the end of therapy, a notable increase in CBE was observed in all corneal sectors, suggesting regrowth of corneal subbasal nerve fibers and effective sensory transmission.

After Cenegermin treatment, improvement in IVCM parameters paired those of the CBE for each peripheral sector, throughout the follow-up. Although the increase in CBE in the central sector was less noticeable, there was a remarkable overall increase in corneal sensitivity at the 8-month follow-up compared to baseline. Some studies reported faster recovery of corneal sensitivity than objective nerve detection after both epi-off crosslinking and penetrating keratoplasty [16, 17]. This was likely due to the inability of the instrument used (slit scanning confocal microscope) in detecting fine regenerating nerves rather than their real absence. Diode-laser IVCM has a higher contrast and a shallower step size in depth compared to slit scanning confocal microscope, which allows better identification of subtle nerves [17]. Nonetheless, the presence of opacities and stromal alterations in NK stage 3 could potentially reduce corneal transparency and visibility of subtle nerves by the IVCM. For the quantification of nerve parameters, we used the CNFL as the density measure, since it takes into account all the fibers detectable in the analyzed frame, and so is more sensitive than the ACCMetrics’s corneal nerve fiber density (CNFD) parameter in quantifying the small fibers seen during nerve regeneration. The same is true for CTBD, which considers all the branch points and not only those emanating from a major nerve trunk, like the corneal nerve branch density (CNBD) parameter does.

Like Mastropasqua et al. [7] the present study found an improvement in corneal sensitivity and subbasal nerves regrowth at the end of treatment; however, in our series, nerve regrowth was not present and not complete in all corneal sectors in all patients at 8 weeks. Moreover, after completion of therapy, CBE and IVCM parameters showed progressive and objective improvement over time with a strong correlation between CBE and all IVCM parameters. We suppose that the early nerve restoration found at the end of therapy was probably adequate to sustain continuous nerve regrowth over time and more extensive reinnervation. This hypothesis is shared by Pan et al. [18], who found that the amount of NGF in the tear fluid after corneal grafting was increased, along with corneal reinnervation. On the other hand, when early nerve restoration is not adequate at the end of therapy, endogenous NGF will decline progressively and corneal surface homeostasis may be lost due to its inadequate production over time. Indeed, corneal surface homeostasis results from the interaction of several factors, among which a pivotal role is played by corneal epithelium and nerves (“epithelium-nerve virtuous circle”). Corneal nerves release trophic factors that modulate the proliferation, differentiation, and integrity of corneal epithelial cells (e.g., acetylcholine, substance P, neuropeptide Y, calcitonine-gene related peptide); and in turn, the corneal epithelium produces factors for stimulating growth, maturation, and survival of nerves (e.g., NGF, brain-derived neurotrophic factor, ciliary neurotrophic factor, neurotrophin 3 and 4/5) [1, 19]. Alterations in any one of these components compromises corneal surface homeostasis. The present study objectively demonstrated the 8 months results on corneal re-epithelialization and nerve regrowth in NK patients treated with Cenegermin and thus restoration of the main elements of the "epithelium-nerve virtuous circle”.

Nerves regrowth was detectable in eyes that presented a healed epithelium regardless of the cause of the NK, nonetheless particularly effective outcomes were found in eyes with NK following penetrating keratoplasty (PK) and even better IVCM results in those secondary to endolaser retinopexy. In these two conditions, trigeminal damage affects the post ganglionic fibers, with the difference that in PK the nerves are transected in both the donor and the recipient cornea, whereas endolaser treatment damages the long ciliary nerve but does not results in its complete interruption [20]. For these reasons, it seems likely that corneal nerve restoration would be harder to achieve in patient with pre-ganglionic trigeminal damage (e.g., patient no. 13).

Relapse occurred in two patients during follow-up. In one (no. 3) new NK developed due to iatrogenic damage. This patient underwent trigeminal ganglion ablation for severe trigeminal postherpetic neuralgia 7 months after the end of Cenegermin treatment. In the second patient (no. 4), exacerbation of ocular GVHD induced severe inflammation that altered the ocular surface and interrupted the restored corneal homeostasis [21]. While nerve regrowth after therapy was present in both patients, a new injury along the nerve pathway occurred and IVCM documented the absence of the previously regrown nerves in both cases and presence of inflammatory cells in the latter. It is conceivable that another cycle of Cenegermin could have been beneficial for these patients.

Our study has some limitations, including the absence of a control group, due to the lack of an effective comparator treatment, and the absence of conjunctival sensation data, which could have added information about the level of the neural damage. The use of a preservative free antibiotic as prophylaxis until the closure of the ulcer could have act as a confounding factor, nonetheless the risk of an infective keratitis outweighs the benefit of avoiding the prophylaxis to reduce potential biases. Moreover, the relatively small sample size and the non-comparative nature of the study preclude definitive deductions.

## Conclusion

Our findings demonstrate corneal re-innervation and recovery of corneal sensitivity at the end of treatment and provide objective evidence that nerve growth and corneal sensitivity continue to increase beyond the end of therapy. Cenegermin treatment boosted restoration of the “epithelium-nerve virtuous circle”, leading to recovery of corneal homeostasis, which maintenance seems essential for long-lasting stability of the ocular surface.

## Data Availability

The datasets used and/or analyzed during the current study are available from the corresponding author on reasonable request.
